# Personalized adherence interventions using medication adherence technologies in polypharmacy management in epilepsy: An interprofessional case report

**DOI:** 10.1016/j.ebr.2025.100767

**Published:** 2025-04-07

**Authors:** Micheline C. Sarbach, Stephan Rüegg, Samuel S. Allemann, Isabelle Arnet

**Affiliations:** a Pharmaceutical Care Research Group, Dept. Pharmaceutical Sciences, University of Basel, Klingelbergstrasse 50, 4056 Basel, Switzerland; b Neurology Clinic, University Hospital Basel, Petersgraben 4, 4031 Basel, Switzerland

**Keywords:** Epilepsy, Medication adherence, Medication adherence technologies, Electronic monitoring, Therapeutic drug monitoring, Interprofessional care, Shared decision making

## Abstract

We describe a male patient with symptomatic multifocal epilepsy struggling with medication adherence. He accepted escalating adherence interventions with conventional dosing aids such as pillboxes and medication adherence technologies (MATech) including a smart medication dispensing and adherence device (SMAD). Adherence metrics and drug levels were continuously monitored by electronic monitoring (EM) and therapeutic drug monitoring (TDM). Initially, adherence metrics with pre-filled punch cards supplied by a community pharmacy showed 64% taking adherence, 63% timing adherence, and 47% correctly dosed days, including a five-day medication holiday. Adherence improved with a pharmacy-filled SMAD, reaching 93% taking adherence, 90% timing adherence, 86% correctly dosed days, and no medication holiday. No epileptic seizures were noticed during periods with EM. The patient was satisfied with the SMAD while struggling with other adherence tools. Adherence tended to decrease once adherence reminders were removed. In conclusion, we emphasize the importance of personalized adherence strategies and shared decision-making. This case highlights the positive impact of an interprofessional adherence improvement program, integrating efforts from researchers, neurologists, and community pharmacists.

## Highlights

• This case report describes clinical findings in a single individual.
• Personalized adherence interventions improved taking adherence from 64% to 93%.
• Adherence tended to decrease once adherence reminders were removed.
• Electronic monitoring in general enables insight into long-term adherence behavior.
• Assessing personal circumstances can help identify strategies to improve adherence.

## 1 Introduction

### 1.1 Adherence

Epilepsy is a neurological condition marked by recurrent seizures, affecting approximately 70 million people worldwide. [1] The treatment with antiseizure medication (ASM) is fundamental and treatment options consist of approximately 30 different active compounds. Because of the short half-life of many ASM, regimens with multiple daily intakes are common, which may negatively affect medication adherence. [2] Determinants of poor adherence are defined as modifiable and non-modifiable factors. [2] While the prescribed medication, patient motivation, beliefs, and knowledge are modifiable, factors such as lifestyle, socio-economic circumstances, and healthcare systems are only conditionally modifiable. Thus, adherence-enhancing interventions should target modifiable factors and should be tailored to non-modifiable factors. [3].

### 1.2 Therapeutic drug monitoring (TDM)

Therapeutic drug monitoring (TDM) of ASM levels represents a standard practice to monitor the course of disease and enables to make assumptions about medication adherence. [4], [5] However, in many cases TDM only confirms recent ASM intake, not long-term adherence, due to the short half-life of many ASM.

### 1.3 Electronic monitoring (EM)

Electronic monitoring (EM) provides supplementary insights to medication adherence and patients’ intake behaviour, due to its accuracy and data reliability. [6], [7] During EM, patients are equipped with medication adherence technologies (MATech). For single medications, these may include devices such as bottles with electronic lids that track usage. For multiple medications, options include button-press sensors with timestamp recording, punch cards with electronic back films, or smart medication dispensing and adherence device (SMAD) that dispense pre-filled blister pouches on a programmed schedule.

### 1.4 Aim

This case report aims to illustrate the interprofessional development of personalized adherence interventions for an individual with epilepsy managing polypharmacy and co-morbidities using MATech.

## 2 Methods

This case is reported in accordance with CARE (consensus-based clinical case reporting guideline). [8] Adherence interventions were based on Demonceau et al.'s eight interventions. [6] We implemented a behavioral-counseling intervention supported by reminder systems and EM. Strategies included reinforcing intake behavior using dosing aids, customizing medication regimens to the patient's daily routine, and conducting a medication review. Community pharmacists simplified the medication regimen, for instance by avoiding tablet splitting. All clinical data and laboratory parameters were extracted from medical reports. All source data were stored in the generators’ local systems (clinic, community pharmacy, monitoring data on the patient’s devices or server until downloaded). All generated data were stored on a password-protected server at the University of Basel. EM data were extracted as csv files and analyzed using Microsoft Excel. Graphs were generated using Microsoft Excel and Power Point. Data obtained from EM were analyzed using established adherence metrics. These include taking adherence, timing adherence, correctly dosed days, and medication holidays, as described in previous studies. [7], [9], [10] Additionally, the average intake times for morning and evening doses, including a ± 25 % grace period, were determined. Informed consent has been obtained from the patient.

## 3 Case report

This case involves a 35 years-old male patient diagnosed at age 10 with symptomatic multifocal epilepsy from bilateral perisylvian polymicrogyria. He also has an emotionally labile personality disorder, likely a co-morbidity of his epilepsy, characterized by impulse control issues, panic attacks, and hallucinatory episodes under excessive stress. The patient was followed for both disorders at the outpatient clinic of a Neurological Clinic by three monthly visits and had access to an emergency doctor if needed. The control of his seizures was difficult given his psychiatric co-morbidity and many drug intolerances. The patient was initially treated with valproic acid, which improved his behavioral symptoms but did not achieve seizure freedom. The addition of lamotrigine led to a reduction of seizures, but caused marked bilateral action tremor in his hands. Levetiracetam and later on brivaracetam, as well as perampanel worsened his behavioral problems, zonisamide and topiramate led to severe adverse events with nausea, and food intolerance. Addition of sodium channel blockers, such as oxcarbazepine, lacosamide, and eventually eslicarbazepine immediately caused unbearable dizziness. The patient was implanted with a vagus nerve stimulator which improved the behavioral difficulties, but not the control of the seizures. At the end, the addition of cenobamate resulted in complete seizure freedom. However, focal non-aware and focal to bilateral tonic-clonic breakthrough seizure clusters occurred resulting in severe injuries, including significant tongue biting up to radius fractures. Despite the patient stated regular medication intake, breakthrough seizures were clearly correlated with ASM drug levels below the therapeutic range. Suspecting non-adherence based on highly variable ASM levels and implausible dose-level relationships the neurologist advised the patient to enroll in a pharmaceutical care service to enhance medication adherence.

**Fig. 1:**
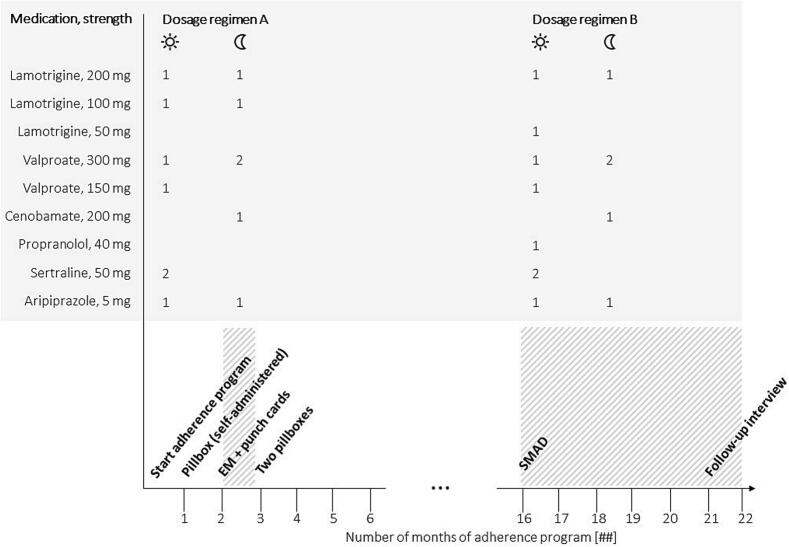
**Medication and adherence intervention schedule:** Antiseizure medication and co-medication with active agent and strength and dosage regimens for morning and evening doses (y-axis) and interventions (x-axis) during the adherence program from month 1 to 22, with dashed areas indicating electronic monitoring (EM). The three dots indicate that the adherence enhancement program was paused during that time. SMAD = smart medication dispensing and adherence device.

The patient, who lived with his family in a suburban area and worked part-time in town, reported smoking approximately one pack of cigarettes per week with no plans to quit. He agreed to join an adherence improvement program involving collaborative efforts between the Pharmaceutical Care Research Group and a local community pharmacy. The patient presented to the pharmacy team with a triple ASM treatment administered in a twice-daily regimen consisting of valproate, lamotrigine, and cenobamate. The co-medication included sertraline and aripiprazole to treat the emotionally labile personality disorder (Fig. 1, dosage regimen A). The patient was using a self-managed pillbox with medications obtained from his community pharmacy.

### 3.1 EM interventions and analysis

**Fig. 2:**
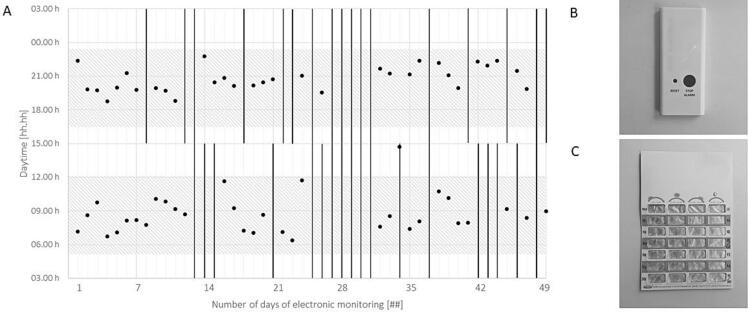
**Electronic monitoring (EM) with punch cards**: The patient took his medication unregularly in the period from month 2 to 3 of the adherence program using **(C)** punch cards and **(B)** a EM device. **(A)** EM was performed by pressing a button indicating medication intake (black dot), grace period (± 25 % around the mean interval; dashed area) and missed doses (black line). One medication holiday of five days started on day 27 of EM.

We thoroughly reviewed all adherence metrics with the patient and implemented interventions consensus-based. The first intervention involved switching from a self-filled pillbox (Dosett®, Karo Pharma, Stockholm, Sweden) to community pharmacy-filled punch cards (Pharmis®, Pharmis GmbH, Beinwil am See, Switzerland) and starting EM by month 2 of the adherence enhancement program. Every Thursday after work, he collected his weekly medications in pre-filled punch cards with 7 x 4 compartments from the community pharmacy in town. Additionally, he was provided with an EM device (Time4Med™, Adherence Innovations, Hong Kong, China) to track each cavity emptied and medication intake by pressing a button. The community pharmacy dispensed a total of 14 punch cards between month 2 and 3 of the adherence enhancement program. The analysis indicated a 64 % taking adherence, 63 % timing adherence and 47 % correctly dosed days over a period of 7 weeks. The mean intake time was 08:43 AM (05:43 AM − 11:43 AM) for morning doses and 08:44 PM (05:44 PM − 11:44 PM) for evening doses. The EM device recorded one medication holiday of five days (Fig. 2). The patient shared that integrating the punch card into his daily routine posed challenges. On weekdays, he left for work at 6:30 AM and was often rushed, causing him to forget the punch cards and miss his morning doses. Moreover, since the punch cards were larger and more noticeable than his previous pillboxes, he felt reluctant taking the medication at work. As a result, in month 3 of the adherence enhancement program, he agreed to switch to using two pillboxes (Dosett®, Karo Pharma, Stockholm, Sweden) on a rotating basis. This meant bringing the empty pillbox to the community pharmacy and picking up a refilled one every Thursday after work.

**Fig. 3:**
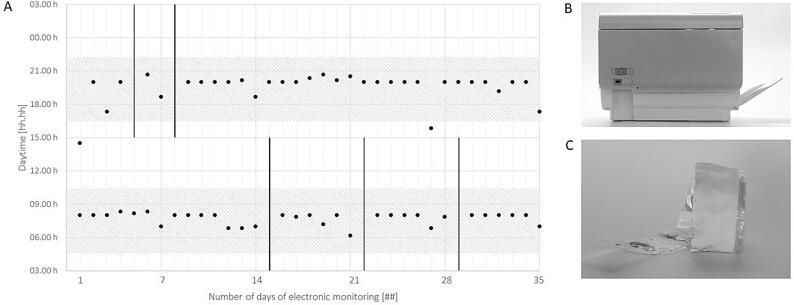
**Electronic monitoring (EM) with a smart medication dispensing and adherence device (SMAD):** The patient took his medication regularly after the implementation of **(B)** SMAD with **(C)** pre-filled blister pouches between month 16 and 17 of the adherence program. **(A)** Electronic monitoring (EM) indicates medication intake (black dot), grace period (± 25 % around the mean interval; dashed area) and missed doses (black line).

The patient’s personal situation degraded suddenly with a divorce, the diagnosis of a severe chronic illness of one of his children, and the resulting emotional instability. The patient continued to exchange pillboxes on Thursdays, but he would not accept EM interventions. In accordance with his neurologist, we paused the adherence enhancement program until a more stable personal situation was achieved. The course of disease was unstable in the following months with epileptic seizures on two following days during month 4 and an epileptic cluster in month 13. During that time, doses of ASM were adapted and co-medication to treat ASM-related tremor was added (propranolol). In month 16 after start of the adherence program, the patient’s social situation stabilised and we resumed the program. The patient accepted to test a SMAD (Medido®, Innospense, Rijswijk, The Netherlands) that delivers pre-filled blister pouches (Medifilm®, Medifilm AG, Oensingen, Switzerland). The investigator (IA) installed the device at the patient’s home, in the kitchen, and programmed daily dispenses at 06:00 AM (two blister pouches) and 08:00 PM (one blister pouch) for his 13 daily pills (Fig. 1, dosage regimen B). A grace period was set at 60 min before the investigator would obtain an email and contact the patient by phone. The patient accepted to pick up a roll of pre-filled blister pouches at his community pharmacy each Thursday after work, and to fill the SMAD manually once back home. The analysis between month 16 and 17 resulted in a 93 % taking adherence, 90 % timing adherence and 86 % correctly dosed days over a period of 5 weeks. Mean intake time was 07:56 AM (04:56 AM − 10:56 AM) for morning doses and 07:40 PM (04:40 PM − 10:40 PM) for evening doses. EM recorded no medication holiday (Fig. 3).

### 3.2 Therapeutic drug monitoring (TDM)

**Fig. 4:**
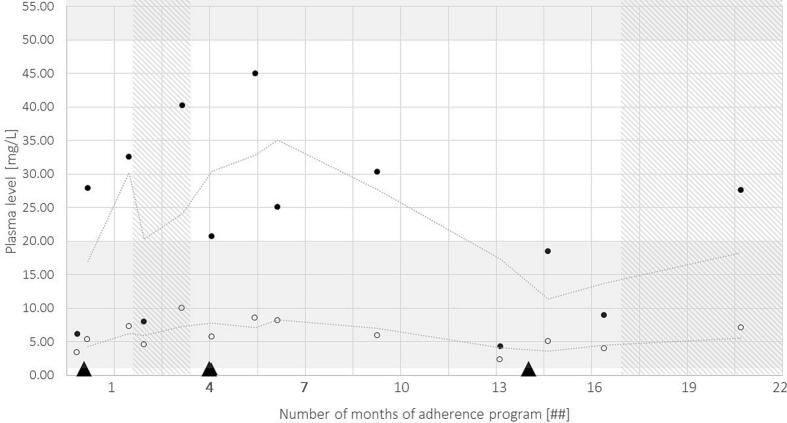
**Therapeutic drug monitoring (TDM):** Serum levels of TDM one month prior and up to month 22 of the adherence program for lamotrigine (white circles; therapeutic range: 1.3 – 20.5 mg/L), valproate (black circles; therapeutic range: 50 – 100 mg/L), with extrapolated serum levels (dotted lines), epileptic seizures (black triangles), and periods of electronic monitoring (EM; dashed area).

TDM was performed for lamotrigine (therapeutic range: 1.3 – 20.5 mg/L) and valproate (therapeutic range: 50 – 100 mg/L) at each consultation at the University Hospital. [11] Valproate serum levels were sub-therapetic during the entire adherence program. During the initial intervention a considerable increase and during the second intervention a slightly overall increase can be observed. Lamotrigine levels remained fairly low and flat throughout the adherence program (Fig. 4). During the challenging personal circumstances and absence of EM from month 6 to 15, trends in decreasing ASM levels were noticeable. The patient reported no epileptic seizures during periods of EM.

### 3.3 Patient-reported device usability and satisfaction

In month 21 of the adherence program, the patient answered open-ended questions on his satisfaction and preferences about the adherence devices. The pillbox received medium satisfaction, being relatively discreet and easy to use. He rated punch cards with EM poorly, finding the blister bulky, indiscreet, and the loose device impractical. The patient rated the SMAD highly, praising its simplicity and flexibility, particularly the ability to manually remove pouch cards, and would recommend it to others. He suggested improvements for EM devices, including a cascading alarm feature with reminders at intervals (e.g., 20 min and one hour) and a reminder function describing the pills' appearance and purpose. He also self-assessed that he achieved the highest level of medication adherence with the SMAD.

## 4 Discussion

This case illustrates the potential of personalized, interprofessional adherence interventions, facilitated by MATech, to enhance medication adherence in patients managing polypharmacy. The remarkable enhancement in adherence from suboptimal levels of 47 % to 64 % with punch cards and EM, to 86 % to 93 % with pre-filled blister pouches delivered by SMAD highlights the importance of tailored adherence strategies. The absence of epileptic seizures during improved adherence periods further supports the clinical relevance of these interventions.

### 4.1 Therapeutic drug monitoring (TDM)

Despite improved adherence and increased overall ASM levels during EM and repeatedly low values before epileptic seizures, lamotrigine TDM levels remained low and valproate levels were highly variable and sub-therapeutic. Valproate exhibits nonlinear pharmacokinetics due to saturable plasma protein binding. This leads to significant variability in the dose-to-plasma concentration relationship among individuals. [5] Lamotrigine exhibits linear pharmacokinetics. However, a large variability in the dose-to-plasma concentration relationship among individuals is described in literature as well, due to its susceptibility to strong drug-drug interactions (DDI). [5] Serum levels around 5 mg/L may be therapeutic for our patient, although it is likely that higher levels may offer greater clinical benefits. [12] Nevertheless, adverse effects limited the increase of dosages of all three ASMs in this specific patient. Further, Inadequate ASM levels may result from variations in metabolism, such as pharmacogenetic (PGx) variation in drug-metabolizing enzymes and transporters. However, PGx testing is currently not recommended for lamotrigine and valproate, because to date known genetic variations are unable to explain sub-therapeutic concentration. [2], [5] PGx studies related to ASM could provide a clearer understanding of the inter-individual variability in ASM levels.

### 4.2 EM interventions

EM was beneficial in this case not only for monitoring adherence, but also for actively enhancing it through reminder functionalities. [13], [14] A Cochrane review from 2022 found that behavioral interventions, including the use of intensive reminders could have a positive impact on ASM in individuals with epilepsy and combined interventions were found to enhance adherence in the intervention groups compared to the control groups. [15] Another factor influencing patient adherence that needs to be considered is the Hawthorne effect, where awareness of being observed alters behavior, underscoring the psychological dimensions of medication adherence. [16] Further research is needed on behavioural-psychological aspects of epilepsy treatment. The observed discrepancy between medication adherence reported by EM and TDM highlights the importance of adopting multimodal approaches to assess adherence and improve treatment outcomes. It should be noted that, in this case, EM did not directly measure medication intake but rather the pressing of a button. With the used EM device, adherence can be both overestimated (button pressed without actual intake) and underestimated (button not pressed despite intake). In contrast, the SMAD device used tends to overestimate adherence, as the button may be pressed to disable the reminder without actual medication intake. EM nonetheless provides a detailed, long-term evaluation of medication intake patterns and adherence behaviors. Targeted interventions focusing on adherence to single medications, rather than overall treatment regimens, could help clarify partially deviated plasma levels.

### 4.3 Interprofessional care

A strength of our approach was the effective communication among stakeholders and the patient’s thorough supervision. For our patient, close coordination among various healthcare professionals was crucial to achieving optimal adherence to ASM. Alongside specialist consultations, low-threshold support from community pharmacies proved highly effective. Community pharmacists consistently monitored the patient’s medication withdrawal and well-being, even during a challenging personal situation. Ideally, community pharmacists would not only oversee adherence but also reassess interventions at each visit and monitor medication adherence. [17] However, we recognize that such a resource- and cost-intensive service cannot be implemented universally. Direct supervision of adherence in the pharmacy setting is only partially feasible, especially in busy urban settings, and mostly limited to directly observed therapy (DOT) for opioid agonist substitution. However, patients could be effectively supported through EM devices, while pharmacies offer targeted counseling as needed. Additionally, the evolution of online portals and virtual consultations (Tele-health) is paving the way for more accessible adherence support.

While SMADs would represent an ideal solution for improving medication adherence, their widespread use remains limited due to several key barriers. Firstly, advanced MATech like SMAD are costly and, in countries such as Switzerland, are currently not reimbursed by health insurance. In contrast, nations such as the Netherlands have already introduced reimbursement for devices such as Medido® (a type of SMAD), facilitating broader access. Moreover, effective use of these tools often relies on pharmacist-led adherence support services, which are not yet consistently reimbursed across healthcare systems. Reimbursement for cost-effective adherence services needs to be established to fairly compensate EM devices and pharmacists for their efforts—ideally covered by health insurance to avoid additional costs to patients. Ongoing clinical studies in pharmaceutical care are promising steps towards enabling the successful and sustainable implementation of such services in community pharmacies. Furthermore, the level of awareness and affinity towards technology among both patients and pharmacies can also play a significant role in determining the adoption of these innovations.

After the end of the adherence program, the patient reported a relapse in to non-adherence due to changes in his life circumstances. These findings are consistent with studies showing that adherence tends to decrease once adherence reminders are removed. [18] Our case report highlights the need for broader research to assess the long-term effectiveness and cost-efficiency of personalized adherence interventions to ensure sustainable integration into healthcare systems and remuneration of healthcare professionals.

## 5 Conclusion

This case underscores the critical role of close interprofessional collaboration, dynamic adaptation to patient life circumstances, and shared decision-making in sustaining medication adherence. Our findings suggest that personalized adherence interventions supported by an interprofessional healthcare team have a positive impact on medication adherence and that restarting interventions at opportune moments can pay off.

## 6 Ethical Statement

We hereby confirm that this manuscript represents original research conducted in accordance with ethical principles and standards for scholarly publishing. All authors have contributed significantly to the work and are accountable for its content. There is no plagiarism, data fabrication, or falsification, and all sources are appropriately cited. The research was conducted with integrity, transparency, and adherence to ethical guidelines, including obtaining informed consent from the patient involved and is available upon request. The study complies with relevant institutional and international ethical standards.

By submitting this manuscript, we affirm our commitment to upholding the ethical standards of scientific publishing and acknowledge the responsibility of all authors in ensuring the validity and integrity of this work. We confirm that we have read the Journal’s position on issues involved in ethical publication and affirm that this report is consistent with those guidelines.

## 7 Declaration of generative AI in scientific writing

During the preparation of this report the authors used ChatGPT (GPT-40) in order to improve the readability and language of the manuscript. After using this tool/service, the authors reviewed and edited the content as needed and take full responsibility for the content of the publication.

## CRediT authorship contribution statement

**Micheline C. Sarbach:** Writing – original draft, Visualization, Validation, Project administration, Methodology, Investigation, Formal analysis, Data curation. **Stephan Rüegg:** Writing – review & editing, Resources, Investigation, Conceptualization. **Samuel S. Allemann:** Writing – review & editing, Supervision, Resources, Funding acquisition. **Isabelle Arnet:** Writing – review & editing, Validation, Supervision, Project administration, Methodology, Investigation, Conceptualization.

## Declaration of competing interest

The authors declare that they have no known competing financial interests or personal relationships that could have appeared to influence the work reported in this paper.
